# Rational use of Xpert testing in patients with presumptive TB: clinicians should be encouraged to use the test-treat threshold

**DOI:** 10.1186/s12879-017-2798-6

**Published:** 2017-10-11

**Authors:** Tom Decroo, Aquiles R. Henríquez-Trujillo, Anja De Weggheleire, Lutgarde Lynen

**Affiliations:** 1 Department of Clinical Sciences, Institute of Tropical Medicine, Nationalestraat 155, 2000 Antwerpen, Belgium; 2 One Health Research Group, Faculty of Health Sciences, Universidad de Las Américas, Quito, Ecuador

**Keywords:** Tuberculosis, Clinical decision-making, Empirical treatment, Molecular diagnostic techniques, Treatment threshold

## Abstract

**Background:**

A recently published Ugandan study on tuberculosis (TB) diagnosis in HIV-positive patients with presumptive smear-negative TB, which showed that out of 90 patients who started TB treatment, 20% (18/90) had a positive Xpert MTB/RIF (Xpert) test, 24% (22/90) had a negative Xpert test, and 56% (50/90) were started without Xpert testing. Although Xpert testing was available, clinicians did not use it systematically. Here we aim to show more objectively the process of clinical decision-making.

First, we estimated that pre-test probability of TB, or the prevalence of TB in smear-negative HIV infected patients with signs of presumptive TB in Uganda, was 17%. Second, we argue that the treatment threshold, the probability of disease at which the utility of treating and not treating is the same, and above which treatment should be started, should be determined. In Uganda, the treatment threshold was not yet formally established. In Rwanda, the calculated treatment threshold was 12%. Hence, one could argue that the threshold was reached without even considering additional tests. Still, Xpert testing can be useful when the probability of disease is above the treatment threshold, but only when a negative Xpert result can lower the probability of disease enough to cross the treatment threshold. This occurs when the pre-test probability is lower than the test-treat threshold, the probability of disease at which the utility of testing and the utility of treating without testing is the same. We estimated that the test-treatment threshold was 28%. Finally, to show the effect of the presence or absence of arguments on the probability of TB, we use confirming and excluding power, and a log10 odds scale to combine arguments.

**Conclusion:**

If the pre-test probability is above the test-treat threshold, empirical treatment is justified, because even a negative Xpert will not lower the post-test probability below the treatment threshold. However, Xpert testing for the diagnosis of TB should be performed in patients for whom the probability of TB was lower than the test-treat threshold. Especially in resource constrained settings clinicians should be encouraged to take clinical decisions and use scarce resources rationally.

With great interest we read the article by Hermans et al. “Treatment decisions and mortality in HIV-positive presumptive smear-negative TB in the Xpert MTB/RIF era: a cohort study” [1]. The authors assessed if clinicians based their clinical decision-making on available Xpert MTB/RIF (Xpert) testing to diagnose tuberculosis (TB) in HIV-positive smear-negative presumptive TB patients. They found that less than half of those diagnosed with smear-negative TB were tested with Xpert MTB/RIF. Hermans et al. concluded that Xpert usage was lower than expected in a setting where Xpert testing is easy accessible. Out of 90 patients who started TB treatment, 20% (18/90) had a positive Xpert test, 24% (22/90) had a negative Xpert test, and 56% (50/90) were started without Xpert testing. The authors concluded that clinicians used Xpert testing as a rule-in test, not that much as a rule-out test, and attribute this to its lower sensitivity in smear-negative HIV-positive patients.

What does this mean, and can this statement be illustrated in a more objective and quantitative manner? Although clinicians are not well acquainted with measures of diagnostic test performance [2], they want to know how much the probability of having a disease is affected by the presence or absence of a clinical sign or a positive or negative test result, like Xpert. Therefore, to show more objectively the process of clinical decision-making, the following important questions require an answer:

- What is the pre-test probability of TB, in smear-negative HIV infected patients with signs of presumptive TB, in this setting?
- At which probability should TB treatment be started (treatment threshold)?
- At which probability should treatment be started without additional testing (test-treat threshold)?
- What is the confirming and excluding power of having a positive or negative Xpert result?
- What is the confirming and excluding power of other clinical arguments?

## Pre-test probability of TB

**Fig. 1:**
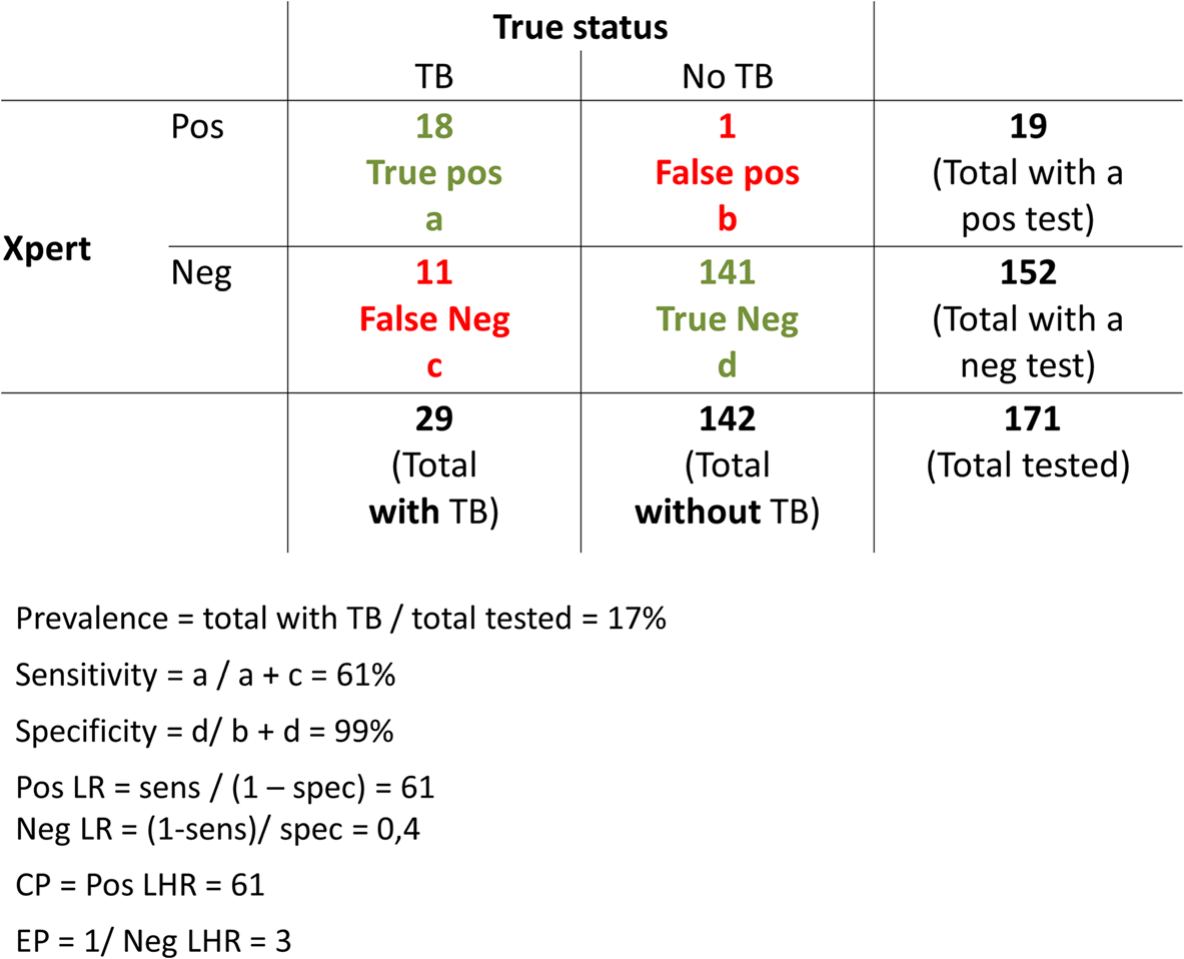
Confirming and excluding power of a positive and negative Xpert test, in smear negative PLHIV with presumptive TB. TB: tuberculosis; Xpert: Xpert MTB/RIF; LR: Likelihood ratio; Pos: positive; Neg: negative; CP: confirming power; EP: excluding power. For a given 61% sensitivity and 99% specificity of Xpert MTB/RIF to detect tuberculosis in smear-negative HIV infected patients with presumptive TB [3], the pre-test probability (prevalence) was 17%, the confirming power of a Xpert result showing “TB detected” was 61 (very strong confirming power), and the excluding power of a Xpert result showing “TB not detected” was 3 (weak excluding power) [13]

The pre-test probability of TB is the prevalence of TB in this subgroup of smear-negative HIV-positive patients. Given that 19 out of 171 individuals tested positive with Xpert, and the 61% sensitivity and 99% specificity of Xpert to diagnose TB in HIV infected persons with a negative smear [3], we can estimate that about 18, 1, 11, and 141 individuals had respectively a true positive, false positive, a false negative, and true negative Xpert result (Fig. 1). Thus, the pre-test probability was probably around 17% (29/171) among those tested with Xpert. Given that TB symptoms, cough, fever, night sweats, weight loss, anorexia, and chest pain did not differ between the two groups, the pre-test probability was likely similar in both groups (those tested and those not tested with Xpert).

The pre-test probability together with the test result influence the post-test-probability of disease.

## Treatment threshold

Another important concept in clinical decision making is the concept of treatment threshold, first developed by Pauker and Kassirer [4]. At what probability of disease is there equipoise between treating and not treating, where is the utility of treating or not treating the same? How many patients are you willing to treat while they do not have the disease (false positives) in order to save 1 who has the disease (true positive). Regardless of the tests available, the minimal level of probability of disease required to treat a patient is called the treatment threshold. This is only influenced by treatment and disease factors. The treatment threshold will be lower when:

- The target disease is severe and/or contagious, like TB.
- The treatment is effective, well tolerated, available, affordable, and acceptable to patients.

Given the relatively high net benefit of treating those who truly have the disease compared to the relatively low risk of treatment in patients with a false positive test result, the threshold is pushed to a lower probability level. In Rwanda, the calculated treatment threshold, based on expected utilities and regret, was 12% [5].

This does not imply that 88% of those started on TB treatment have no tuberculosis. The probability of disease follows a bimodal distribution [6]. In fact, most patients will have either a low or a high probability of disease, only few will be around the treatment threshold [7]. In Uganda, the treatment threshold has not been calculated, but it is unlikely to differ much from the one calculated in Rwanda. If anything, the treatment threshold would be even lower, because of the fact that all patients in the Uganda study are HIV-positive.

Interestingly, Hermans et al. inform the reader that clinicians had to indicate, prior to ordering an Xpert test, if they already had enough arguments to start TB treatment. In other words, they indicated if they had surpassed the treatment threshold [1]. Given the 17% pre-test probability, empirical TB treatment seems justified in this patient population [8]. One could argue that the threshold has been reached without even considering additional tests. When then is it justified to still test with Xpert, even when the probability of disease is above the treatment threshold?

## Test-treat threshold

**Fig. 2:**
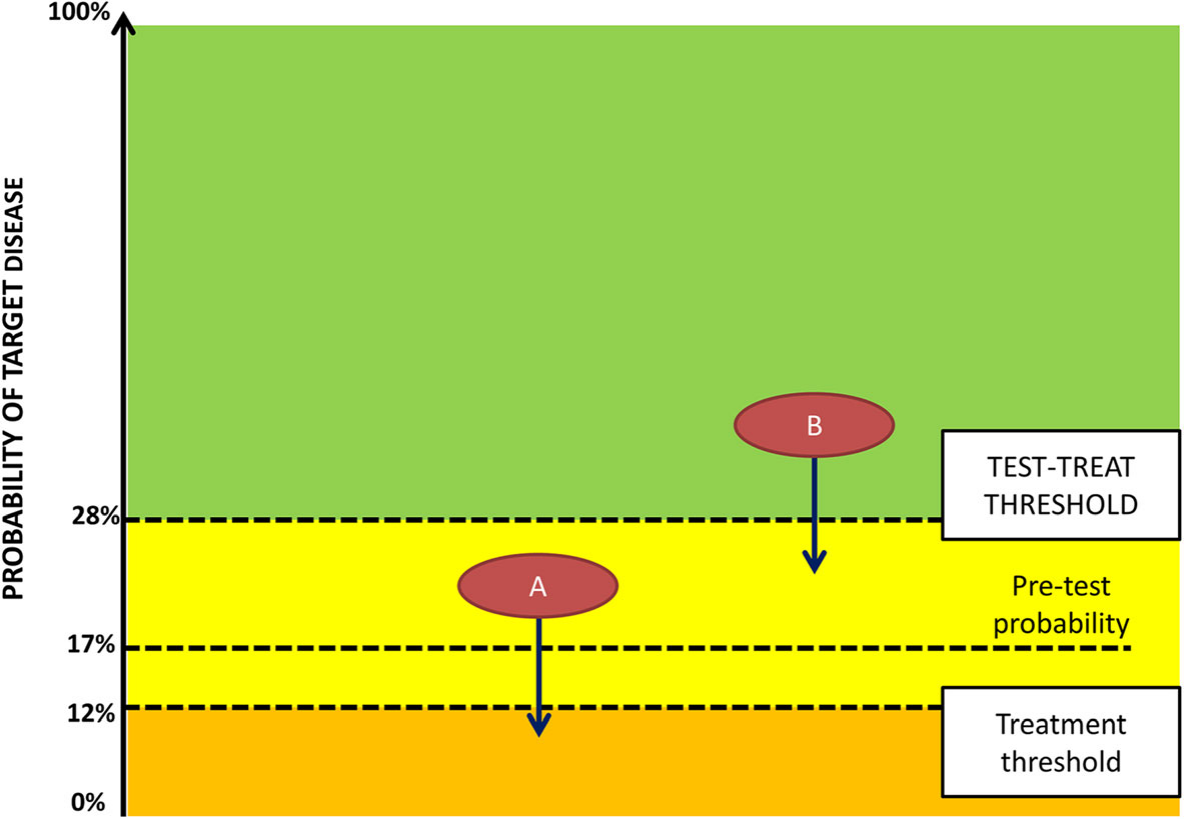
The effect of a negative Xpert result on post-test probability and decision to treat, applied to HIV infected patients with smear-negative presumptive TB, in Uganda. For patient A, with a pre-test probability above the treatment threshold, but below the test-treat threshold, a negative Xpert results in withholding TB treatment. In Patient B, with a pre-test probability above the test-treat threshold, a negative Xpert result has no effect on the decision to treat, as the post-test probability is still higher than the treatment threshold. Thus, when the pre-test probability of the target disease is higher than the test-treat threshold, there is no need to test. The pre-test probability of TB was 17% in this setting. As the treatment threshold was not yet calculated for Uganda, we use in this example the 12%, calculated in Rwanda [5]. The test-treat threshold is the probability of disease at which there is no difference between testing and treating without testing. The distance between the two thresholds depends on the excluding power of negative test result. Given the weak power of negative Xpert result, the test-treat threshold is 28% (12% corresponds to an odds of 0.14, and a log_10_ odds of −0.9. For a weak power-level 0.5 is added, on a log_10_ odds scale. -0.4 log_10_ odds corresponds with a 0.4 odds or 28% test-treat probability. The use of the log_10_ odds scale has been published elsewhere [13])

When you have a good diagnostic test you may consider three options: 1) to treat without testing, 2) to not treat and not test, and 3) to do a test first. This is the threshold approach to clinical decision making [9]. The test-treat threshold is the probability of disease at which the utility of testing and the utility of treating without testing is the same. A final, additional test is only useful when it can raise or lower the probability of disease enough to cross a treatment threshold. When a negative test result will not bring the probability of disease below the treatment threshold, treatment is started without further testing. This occurs when the pre-test probability is higher than the test-treat threshold (Fig. 2) [7, 9].

The test-treat threshold is determined not only by disease and treatment factors (treatment threshold) but also by the excluding power of a negative test. We estimated that the test-treat threshold was 28% (Fig. 2).

## Confirming and excluding power of an argument

The confirming and excluding power of an argument are derived from likelihood ratios (LR), which are often encountered in papers on diagnostic accuracy. Unfortunately, LR are interpreted in different directions: positive LR range between 1 and infinity whereas negative LR range between 1 and 0 [10]. The further from 1, the more the argument has an effect on the probability of the disease [11]. Thus, an argument with a negative LR of 0.01 has more power than an argument with a negative LR of 0.1, which seems counterintuitive [10]. More logic and easier to understand are confirming and excluding power, which are expressed as an absolute number. The higher the numeric value, the higher the power of the argument to confirm or exclude the disease.

- The confirming power is the same as a positive LR, it expresses how many times more likely is a positive test result in a diseased compared to a non-diseased person, and is calculated as the sensitivity divided by (1 – specificity).
- Excluding power is the inverse of a negative LR, it expresses how many times more likely is a negative test result in a non-diseased compared to a diseased person, and is calculated as the specificity divided by (1 – sensitivity).

Values from 2 to 5 result in small changes in the post-test probability of the disease, from 5 to 10 moderate changes, and above 10 large changes [11].

## Confirming and excluding power of Xpert results

Figure 1 shows the different measures, using data provided by Hermans et al. [1], and the sensitivity and specificity of Xpert testing in PLHIV with a negative sputum smear [3].

Indeed, an Xpert result showing “TB detected” has a confirming power of 61, and is a very strong argument in favour of TB diagnosis. Conversely, an Xpert result showing “TB not detected” has an excluding power of 3, and can be considered as a weak argument against diagnosing TB [11]. To use Xpert as a rule-in test, but not a rule-out test, is thus well justified in this study population.

## Combining confirming and excluding power of other clinical arguments

Most TB diagnoses were made without Xpert testing. The article doesn’t provide a lot of data on the arguments that informed the clinical decisions made [1]. Still, the authors mention that the chest X-rays (CXR) showed abnormalities in respectively 69% and 54% of patients with and without an Xpert result. In addition, signs of extrapulmonary TB were present in respectively 17% and 50% of patients with and without an Xpert result [1].

From Cain et al. we learn that the presence of an abnormal CXR has a weak confirming power of 4 (positive LR = 4.42), and a weak excluding power of 2 (negative LR = 0.41) [12]. Lymphadenopathy (any location), which can be interpreted as a clinical sign of extrapulmonary TB in patients with presumptive TB, has a weak confirming power of 3 (positive LR = 3.19), and an excluding power of 1 (negative LR = 0.77) [12]. Values close to 1 are useless for clinical decision-making [11].

**Fig. 3:**
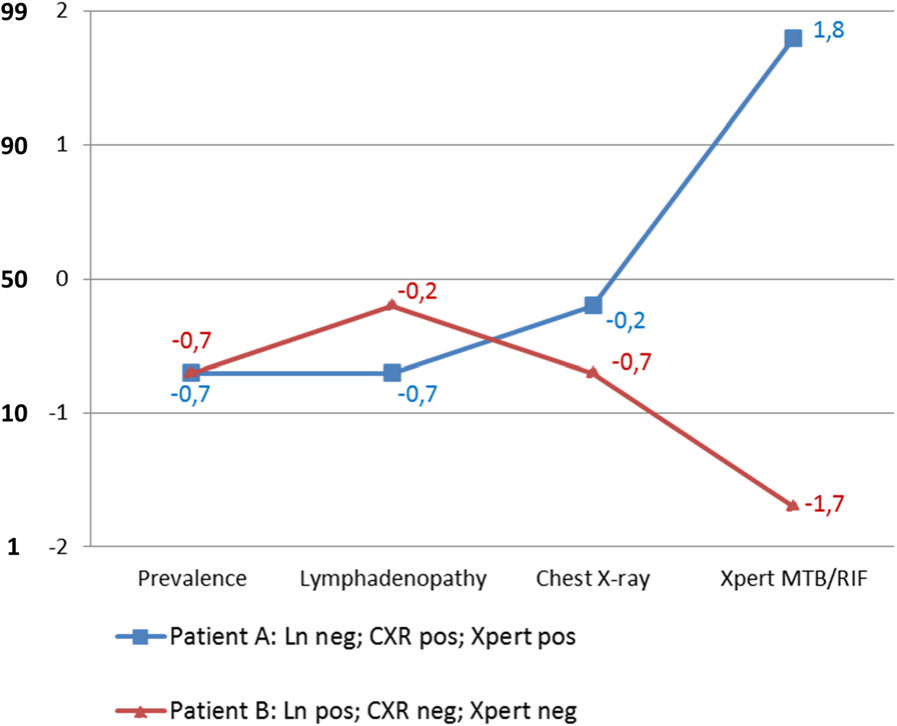
Effect of confirming and excluding power of clinical arguments on the post-test probability of TB, shown on a base-10 logarithmic scale and the corresponding probability scale. Ln: lymphadenopathy; pos: positive; neg: negative. The figure shows two Y-axis: one axis shows the log10 odds scale and the second axis shows the corresponding probabilities. Very strong, strong, good, or weak confirming power allows to advance, respectively, 2, 1.5, 1, 0.5 or 0 steps upward on the log_10_ odds scale, and thus results in a higher post-test probability. Similarly, the excluding power of an argument allows to regress downward and thus results in a lower post-test probability. Patient A presented without lymphadenopathy, had a positive CXR, and a positive Xpert. The pre-test probability (prevalence) was 17%. When converted to an odds, this equals 0.2. The log_10_ of that odds is −0.7. The absence of lymphadenopathy has an excluding power of 1, thus has no effect on the probability. Signs of TB on a CXR has weak confirming power: move 0.5 step upward: −0.7 + 0.5 = −0.2). A positive Xpert has very strong power: −0.2 + 2 = 1.8. After converting 1.8 log_10_ odds to odds, then to a probability, this patient has about 98% post-test probability of TB. The use of the log_10_ odds scale has been published elsewhere [13]. Patient B presented with lymphadenopathy, had a negative CXR, and a negative Xpert. In this patient the post-test probability of TB is about 2%

To show the effect of the presence or absence of these arguments on the probability of TB, and how arguments can be combined, we use a log_10_ odds scale. The mathematical background of this approach has been published elsewhere [13]. Moreover, to simplify the use of the scale, the confirming and excluding power of arguments is categorized into very strong, strong, good, weak, or useless (Table 1) [13]. Figure 3 shows how the probability of TB moves upward or downward on a log_10_ odds scale, as arguments are present or absent in two hypothetical patients.

**Table 1:** Categories for confirming and excluding power, and the corresponding effect on moving toward a higher or lower post-test probability on the log_10_ odds scale

Power of the argument^a^	Strength	Steps on the scale^b^
100 (58–200)	Very strong	2
33 (17 to 57)	Strong	1.5
10 (6 to 16)	Good	1
3 (2 to 5)	Weak	0.5
1	Useless	0

^a^Confirming or excluding power can range between 1 and infinity. However, power is rarely 1000 or more

^b^If confirming power, add the respective number of steps, if excluding power, subtract steps (unit in log_10_ odds)

## Test or treat?

As illustrated in Fig. 2, if the pre-test probability is above the test-treat threshold, empirical treatment is justified, because even a negative Xpert will not lower the post-test probability below the treatment threshold.

**Fig. 4:**
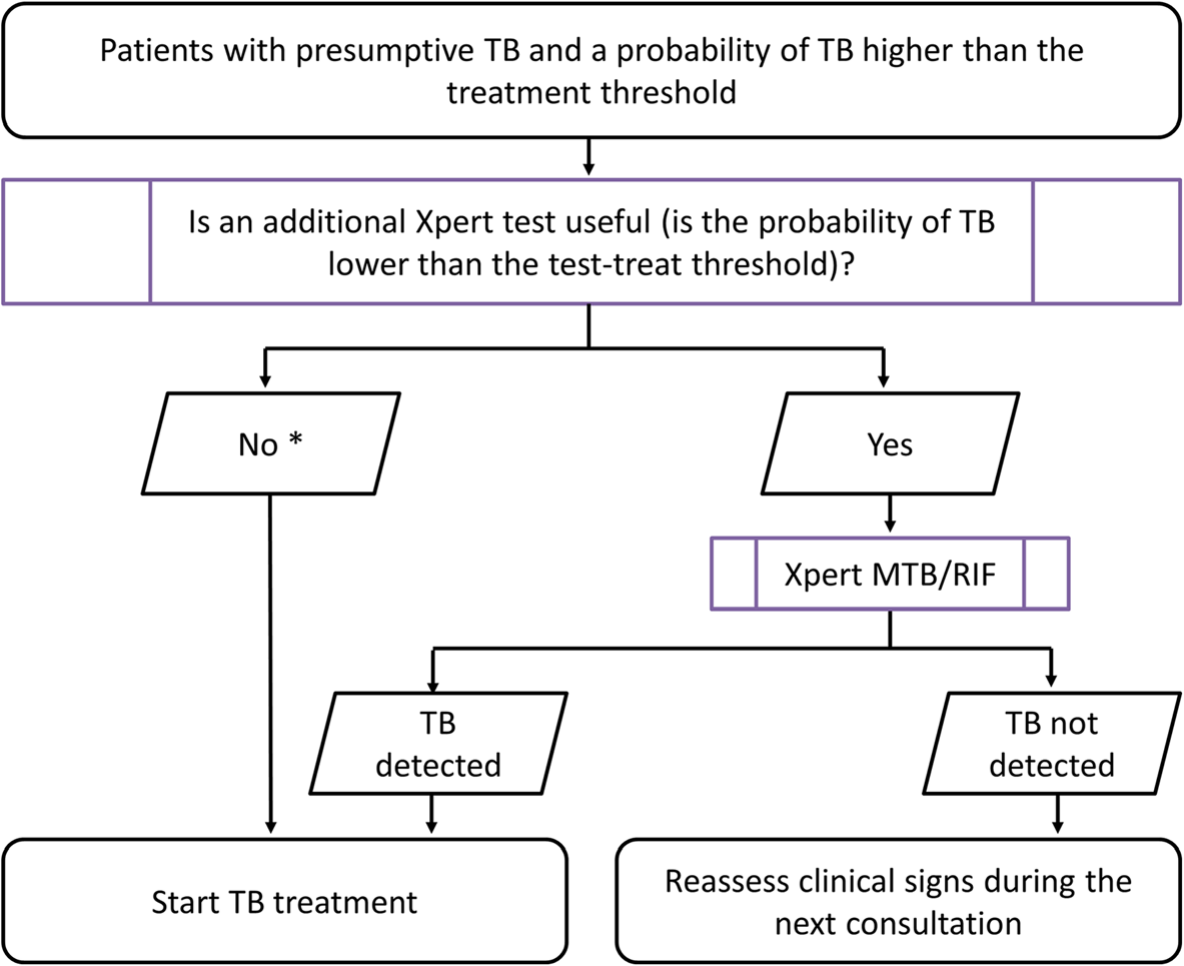
Decision tree for doing an additional test, Xpert MTB/RIF, in patients with a probability of having TB above the treatment threshold. TB: tuberculosis. * In patients with a probability of TB higher than the test-treat threshold, Xpert MTB/RIF testing may still be indicated to detect rifampicin-resistance

Based on the reasoning shown in the paragraphs here above, Xpert testing for the diagnosis of TB should only have been performed in patients for whom the probability of TB was lower than the test-treat threshold [7–9] (Fig. 4).

In case Xpert is done anyway for reasons of diagnosing rifampicin resistance, or because guidelines prescribe it for people living with HIV, a negative result at such high post-test probabilities should not make the clinician doubt to treat the patient anyway for TB [8].

In settings with a lower pre-test probability of having TB, as is often the case in high-income countries, and where there are no financial barriers in using Xpert testing, testing is always justified in patients with presumptive TB. Whether a negative Xpert allows to exclude TB as a diagnosis depends on the treatment threshold established in these settings, which may differ slightly from resource-poor settings.

In conclusion, this example illustrates well the process of clinical decision-making. Especially in resource constrained settings clinicians should be encouraged to take clinical decisions and use scarce resources rationally. Moreover, clinical decision-making is nor linear or static, rather an iterative process. Every follow-up consultation the clinician will re-assess the patient. New signs may emerge, and thus alter the clinical decision-making process. The described structured and objective approach may not be feasible to conduct during every clinical consultation, however, the concepts of confirming and excluding power and test-treat threshold may provide structure to the clinician when ruling in or out a diagnosis.

## Abbreviations

CXR: chest X-ray
LR: likelihood ratio
TB: tuberculosis
Xpert: Xpert MTB/RIF
